# Mutant TP53 interacts with BCAR1 to contribute to cancer cell invasion

**DOI:** 10.1038/s41416-020-01124-9

**Published:** 2020-11-04

**Authors:** Alvin Kunyao Guo, Yoko Itahana, Veerabrahma Pratap Seshachalam, Hui Ying Chow, Sujoy Ghosh, Koji Itahana

**Affiliations:** 1 Cancer and Stem Cell Biology Programme, Duke-NUS Medical School, 8 College Road, Singapore, 169857 Singapore; 2 Centre for Computational Biology, Duke-NUS Medical School, 8 College Road, Singapore, 169857 Singapore; 3 School of Applied Science, Temasek Polytechnic, 21 Tampines Avenue 1, Singapore, 529757 Singapore

**Keywords:** Oncogenes, Metastasis

## Abstract

**Background:**

Mutant TP53 interacts with other proteins to produce gain-of-function properties that contribute to cancer metastasis. However, the underlying mechanisms are still not fully understood.

**Methods:**

Using immunoprecipitation and proximity ligation assays, we evaluated breast cancer anti-estrogen resistance 1 (BCAR1) as a novel binding partner of TP53^R273H^, a TP53 mutant frequently found in human cancers. The biological functions of their binding were examined by the transwell invasion assay. Clinical outcome of patients was analysed based on *TP53* status and *BCAR1* expression using public database.

**Results:**

We discovered a novel interaction between TP53^R273H^ and BCAR1. We found that BCAR1 translocates from the cytoplasm into the nucleus and binds to TP53^R273H^ in a manner dependent on SRC family kinases (SFKs), which are known to enhance metastasis. The expression of full-length TP53^R273H^, but not the BCAR1 binding-deficient mutant TP53^R273H^Δ102–207, promoted cancer cell invasion. Furthermore, among the patients with mutant *TP53*, high *BCAR1* expression was associated with a poorer prognosis.

**Conclusions:**

The interaction between TP53^R273H^ and BCAR1 plays an important role in enhancing cancer cell invasion. Thus, our study suggests a disruption of the TP53^R273H^–BCAR1 binding as a potential therapeutic approach for TP53^R273H^-harbouring cancer patients.

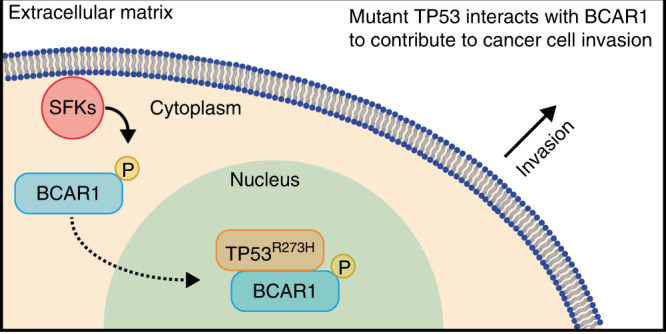

## Background

The transcription factor TP53 is one of the most important tumour suppressors. TP53 prevents tumour development and progression by regulating target genes involved in cell cycle arrest, senescence, apoptosis, autophagy, metabolic reprogramming, tumour microenvironment signalling, and the suppression of cancer cell migration and invasion.^1–7^ Given its central role in tumour suppression, it is not surprising that the TP53 pathway is frequently inactivated through mutations in TP53 itself or alterations in its regulators including CDKN2A (also known as p14ARF) and MDM2.^8^ In contrast to other tumour suppressors such as RB1, APC, NF1, NF2, and VHL, where deletion or nonsense mutations result in little or no protein expression of these tumour suppressors, most TP53 mutations are missense mutations that produce stable and long-lived proteins. A number of hotspot mutations including R273H are identified within the DNA-binding domain of TP53.

Mutation within the DNA-binding domain often leads to the loss of DNA-binding ability of TP53. Therefore, such mutant TP53 is unable to induce *CDKN1A*, *BBC3/PUMA* and *BAX* to trigger tumour-suppressive functions, and the mutations in *TP53* have been known to be similar with deletion of *TP53*, often referred to as the loss-of-function of TP53.^4,5^ However, in vivo knock-in mice harbouring mutant forms of *Trp53* (mouse orthologue corresponding to human *TP53*) developed more metastatic tumours as compared to *Trp53*-null mice.^9,10^ *TP53* is mutated more in the advanced stage of human cancers, compared to the early stages, suggesting that mutant TP53 is involved in metastasis in human cancer as well.^11,12^ These findings shifted the paradigm of understanding of TP53 mutations. In addition to the loss-of-function properties, mutant TP53 have oncogenic gain-of-function properties often related to invasion or metastasis;^13^ for example, they can promote epithelial-to-mesenchymal transition (EMT),^14,15^ disrupt focal adhesion signalling pathways,^16^ augment filopodia formation,^17^ disrupt mammary tissue architecture,^18^ and promote integrin or growth factor receptor recycling.^19,20^ Several mechanisms are proposed through which mutant forms of TP53 exhibit their gain-of-function properties: (i) binding to proteins such as transcription factors or cofactors to repress or augment their activities, (ii) binding to other proteins to modulate their activities, and (iii) binding to certain DNA structural motifs.^8,13,21–24^ However, the gain-of-function properties of mutant TP53 are still not fully understood due to their cell-type and cell-context dependency regulated by multiple underlying mechanisms.^13,25,26^

To gain novel insights into these mechanisms, we looked for interacting partners of TP53^R273H^ and identified BCAR1 as a novel mutant TP53^R273H^ binding protein. We propose that the formation of the TP53^R273H^–BCAR1 complex is a novel mechanism of mutant TP53-mediated gain-of-functions for cancer cell invasion.

## Methods

### Cell lines, cell culture, and reagents

MDA-MB-468, A431, H1299, and HEK 293T cells were obtained from the American Type Culture Collection (ATCC, LGC Standards, Teddington, UK). U251MG cell line was purchased from CLS Cell Lines Service GmbH (Eppelheim, Germany). All cell lines were cultured in Dulbecco’s modified Eagle medium (DMEM, Thermo Fisher Scientific, Waltham, MA, USA) supplemented with 10% foetal bovine serum (FBS) (Thermo Fisher Scientific) and maintained in a 37 °C incubator with 5% CO_2_. SRC family kinase inhibitors (SFKs) CGP77675 and PP2 were purchased from Axon Medchem (Reston, VA, USA) and Sigma Aldrich (St. Louis, MO, USA), respectively. Leptomycin B was purchased from LC laboratories (Woburn, MA, USA). Matrigel was purchased from Corning (Corning, NY, USA). 4',6-diamidino-2-phenylindole (DAPI) was purchased from Nacalai Tesque (Nakagyo-ku, Kyoto, Japan).

### Plasmids

To generate human BCAR1 expression constructs, full-length *BCAR1* complementary DNA (cDNA) was amplified by RT-PCR using cDNA from MDA-MB-468 cells as a template. PCR products were cloned into pcDNA3.1(+)-FLAG3, pcDNA3.1(+)-MYC3, or pCMV-MYC3 vector backbones. The CMV-TP53^R273H^ plasmid was generated by site-directed mutagenesis PCR using CMV-TP53^WT^ as a template, which was kindly provided by Dr. Yanping Zhang (University of North Carolina at Chapel Hill, USA). Truncation mutants of TP53^R273H^ (1–292, 102–393, and 293–393) were generated by PCR cloning, and deletion mutants of TP53^R273H^ (Δ102–207 and Δ208–292) were generated by site-directed mutagenesis PCR using CMV-TP53^R273H^ as a template. Cas9-EGFP was obtained from Addgene (Watertown, MA, USA), and the plasmid containing gRNA targeting *TP53* was constructed as previously described.^27^ ScreenFect A (FUJIFILM Wako Pure Chemical Corporation, Osaka, Japan) or calcium phosphate transfection method was used for plasmid transfection according to the manufacturer’s instructions or as previously described,^28^ respectively. When FLAG- or MYC-tagged TP53^R273H^ (or BCAR1) plasmid was transfected, we used the plasmid containing FLAG or MYC sequence as a negative control.

### Indirect immunofluorescence analysis

H1299 cells were fixed with 4% paraformaldehyde in phosphate buffered saline (PBS), permeabilised with 0.2% Triton-X, and blocked with 5% bovine serum albumin (BSA) in PBS. Mouse anti-BCAR1 (1:1000, 21/p130[Cas], BD Biosciences, San Jose, CA, USA), rabbit anti-TP53 (1:1000, FL393, Santa Cruz Biotechnology, Dallas, TX, USA) and mouse anti-FLAG (1:1000, M2, Sigma Aldrich) were used as primary antibodies. Alexa Fluor 488- or Alexa Fluor 594-conjugated donkey anti-mouse IgG and Alexa Fluor 488- or Alexa Fluor 594-conjugated donkey anti-rabbit IgG (1:1000, Jackson ImmunoResearch, West Grove, PA, USA) were used as secondary antibodies. DAPI was used for staining the nuclei. Images of the nuclei, BCAR1, and TP53^R273H^ were acquired using 405, 488, and 591 nm lasers on a confocal microscope (Carl Zeiss LSM 710, Oberkochen, Germany) equipped with oil-immersion objective lens (NA 1.40, ×63; Plan Apochromat, Carl Zeiss) and ZEN 2010 software (version 6.0.0.486; Carl Zeiss). Images were cropped using ImageJ software (version 1.45f; National Institutes of Health, Bethesda, MD, USA).

### Co-immunoprecipitation

Cells were washed three times with PBS and lysed with ice-cold 0.1% NP40 lysis buffer (50 mM Tris-HCl [pH 7.5], 0.1% NP40, 150 mM NaCl, 50 mM NaF, 1 mM phenylmethylsulfonyl fluoride, 1 mM Na_3_VO_4_, and protease inhibitors). All subsequent steps were performed at 4 °C. Cell lysates were pre-cleared with 50 µL of Sepharose CL-4B beads (Sigma Aldrich) for 30 min, and 1 mg of total proteins were immunoprecipitated with 1 µg of the indicated antibodies (rabbit or goat anti-TP53 [FL393, Santa Cruz Biotechnology], mouse anti-FLAG [M2, Sigma Aldrich], mouse anti-MYC [9E10, Santa Cruz Biotechnology], or mouse anti-BCAR1 [21/p130[Cas], BD Biosciences]) for 16 h. After incubating the lysates with 10 µL of 50% Protein A/G agarose beads slurry (Thermo Fisher Scientific) for 2 h, the agarose beads were washed three times using ice-cold 0.1% NP40 lysis buffer before being subjected to SDS-polyacrylamide gel electrophoresis and protein analysis.

### Proximity ligation assay and quantification of proximity ligation foci

Proximity ligation assay (PLA) was performed according to the manufacturer’s instructions (Sigma Aldrich). Briefly, H1299 or U251MG cells seeded onto µ-dish (ibidi, Grafelfing, Germany) were fixed with 4% paraformaldehyde in PBS. Cells were then permeabilised using 0.2% Triton-X and blocked with 5% BSA in PBS. For H1299 cells transfected with CMV-TP53^R273H^ and/or pCMV-MYC3-BCAR1, mouse anti-TP53 (1:1000, DO-1, Santa Cruz Biotechnology) and rabbit anti-MYC (1:1000, Proteintech Group, Inc, Rosemont, IL, USA) were used as primary antibodies. For H1299 cells transfected with pcDNA3.1(+)-TP53^R273H^-FLAG3, TP53^R273H^ (1–292)-FLAG3, and/or pcDNA3.1(+)-MYC3-BCAR1, mouse anti-FLAG (1:1000, M2, Sigma Aldrich) and rabbit anti-MYC (1:1000, Proteintech Group, Inc) were used as primary antibodies. For U251MG cells, rabbit anti-TP53 (1:1000, FL393, Santa Cruz Biotechnology) and mouse anti-BCAR1 (1:1000, 21/p130[Cas], BD Biosciences) were used as primary antibodies. Anti-mouse PLUS and anti-rabbit MINUS probes (1:500, Duolink, Sigma Aldrich) were used as secondary antibodies. Ligation and amplification reactions were performed according to the manufacturer’s instructions. To confirm the localisation of TP53^R273H^-FLAG3 and TP53^R273H^ (1–292)-FLAG3, cells were also stained with Alexa Fluor 594-conjugated donkey anti-mouse IgG and CF680R-conjugated donkey anti-rabbit IgG (1:1000, Biotium, Fremont, CA, USA) as secondary antibodies after PLA assay. Cells were then washed, and the nuclei were stained with DAPI. Images of the nuclei, proximity ligated foci, TP53^R273H^, and BCAR1 were acquired using 405, 488, 591, and 688 nm lasers on a confocal microscope (Carl Zeiss LSM 710) equipped with oil-immersion objective lens (NA 1.40, ×63; Plan Apochromat, Carl Zeiss) and ZEN 2010 software (version 6.0.0.486; Carl Zeiss). Images were cropped using ImageJ software (version 1.45f; National Institutes of Health). The fluorescence intensity of proximity ligated foci was measured by ImageJ software using 8-bit images. Briefly, the tracing tool was used to outline individual cells, and the ‘measure’ function was used to obtain mean fluorescence intensity of each proximity ligated foci. Total fluorescence intensity in each cell was normalised by cell area and plotted as a dot in the graph. Statistical analysis of data was done by unpaired Student’s two-sided *t* test.

### Three-dimensional spheroid invasion assay and quantification of the invasion area

U251MG cells (1 × 10^3^) were seeded into ultra-low attachment 96-well round-bottom plates (Corning) containing 200 µL of DMEM supplemented with 10% FBS and cultured for 2 days to form spheroids. Following spheroid formation, 100 µL of DMEM was replaced with 100 µL of 100% Matrigel (Corning) and incubated at 37 °C for 1 h before overlaying with 100 µL of DMEM containing 10% FBS. This was set as day 0. Micrographs of the spheroids were taken at ×10 magnification using an inverted Phase Contrast microscope (IX73, Olympus, Tokyo, Japan) coupled with a DP71 camera (Olympus). The area of cells invading into Matrigel was measured by ImageJ software using 8-bit images of spheroids. Briefly, the ‘find edges’ function with a minimum and a maximum threshold value of 0 and 50, respectively, was applied to each spheroid image. The tracing tool was then used to outline the spheroids. Area of spheroids measured on day 0 was subtracted from the area of spheroids measured on day 2 to obtain the area of cells invading into Matrigel. The data are presented relative to the sh*Control* cells.

### Transwell invasion assay

Matrigel (Corning) diluted in serum-free DMEM was coated on the upper side of 8 μm pore-size transwell inserts (Corning) and allowed to dry. MDA-MB-468 (1 × 10^5^), U251MG (1 × 10^5^), or A431 (1 × 10^6^) cells suspended in serum-free DMEM were then seeded onto the Matrigel-coated transwell inserts (Corning) and placed into 24-well plates containing DMEM supplemented with 20% FBS. Cells were allowed to invade across the transwell insert for 20 h (MDA-MB-468 and A431 cells) or 12 h (U251MG cells) before fixing and permeabilising with 4% paraformaldehyde and 0.1% Triton-X, respectively. Non-invading cells in the upper side of the transwell were removed by cotton-tipped swab before staining with 0.1% Crystal Violet solution (Sigma Aldrich). The excess stain was washed with water, and the inserts were allowed to dry. More than five micrographs per sample were taken at ×11.5 magnification using a dissection microscope (Olympus) coupled with a DP71 camera (Olympus). The area of invasion was measured by ImageJ software using 8-bit images of micrographs. Briefly, the ‘analyse particles’ function was applied to each transwell image. The data are presented as a percentage. Statistical analysis of data was done by unpaired Student’s two-sided *t* test.

### Protein analysis

Cells were washed three times with PBS and lysed with 2% SDS lysis buffer (2% SDS, 50 mM Tris-HCl [pH 6.8], 10% glycerol). Protein concentration was determined using DC Protein Assay Reagents (BioRad Laboratories, Hercules, CA, USA). Equal amounts of total protein were separated by SDS-polyacrylamide gel electrophoresis and transferred to nitrocellulose membranes. Membranes were blocked with 5% non-fat milk in PBS containing 0.1% Tween-20 and blotted with the indicated antibodies (mouse anti-TP53 [1:1000, DO-1, Santa Cruz Biotechnology], rabbit anti-TP53 [1:1000, FL393, Santa Cruz Biotechnology], mouse anti-BCAR1 [1:1000, 21/p130[Cas], BD Biosciences], mouse anti-ACTIN [1:50,000, C4, Merck Millipore, Darmstadt, Germany], rabbit anti-phospho-p130 Cas/BCAR1 (Tyr165) [1:1000, Cell Signaling Technology, Danvers, MA, USA], mouse anti-FLAG [1:1000, M2, Sigma Aldrich], rabbit anti-FLAG [1:1000, Proteintech Group, Inc], or mouse anti-MYC [1:1000, 9E10, Santa Cruz Biotechnology]). Detection of blots was done with West Pico (Thermo Fisher Scientific) for secondary antibody conjugated with HRP (1:10,000, HRP-conjugated goat anti-mouse or anti-rabbit IgG, Jackson ImmunoResearch), or done with Odyssey Infrared Imaging System (LI-COR Biosciences, Lincoln, NE, USA) for fluorescent-labelled secondary antibody (1:10,000, DyLight 680- or 800-conjugated goat anti-mouse IgG or DyLight 680- or 800-conjugated goat anti-rabbit IgG, Thermo Fisher Scientific).

### Affinity purification mass spectrometry, CRAPome, and Ingenuity Pathway Analyses

U251MG cells were transduced with empty vector control- or TP53^R273H^-FLAG-expressing adenovirus at large-scale. Cells were lysed with ice-cold 0.1% NP40 lysis buffer and subjected to co-immunoprecipitation using EZview Red anti-FLAG M2 Affinity Gel (M2, Sigma Aldrich). Proteins bound to the beads were eluted with 3xFLAG peptide (Sigma Aldrich), digested with trypsin, and then applied to a C18 column (Thermo Fisher Scientific). Bound proteins were washed with ultrapure water to remove any salt, eluted with acetonitrile, and concentrated using a SpeedVac vacuum concentrator (Thermo Fisher Scientific). Samples were analysed by LC-MS/MS. MS data were analysed using GPM software (http://gpmdb.thegpm.org) with default parameter settings. Proteins with the expectation values log(e)^29^ of −1.0 or lower were used for downstream analysis. A hundred and fifty-one proteins were identified after background subtraction. BioGRID (https://thebiogrid.org/) and PubMed were used to assess if the identified proteins are novel or reported. Identified proteins were queried on CRAPome database^30^ (http://www.crapome.org), and 62 proteins with CRAPome value < 15/411 were considered as specific TP53^R273H^ binding candidates. These were annotated and analysed for molecular and cellular functions through the use of Ingenuity Pathway Analysis^31^ (QIAGEN Inc., https://www.qiagenbioinformatics.com/products/ingenuity-pathway-analysis).

### Kaplan–Meier plots

We downloaded the clinical datasets of Molecular Taxonomy of Breast Cancer International Consortium (METABRIC) for breast cancer and The Cancer Genome Atlas (TCGA) PanCancer Atlas for lung adenocarcinoma from cBioportal database^32,33^ (https://www.cbioportal.org/). The data were sequenced on Illumina platform. We retrieved the RSEM normalised counts for *BCAR1* gene along with *TP53* mutation status (wild type or mutant), survival months, and status (alive or dead). After filtering off samples with missing information, METABRIC dataset has 1904 samples composed of *TP53* mutated (*n* = 659) and *TP53* wild type (*n* = 1245). TCGA PanCancer Atlas dataset has 501 samples composed of *TP53* mutated (*n* = 260) and *TP53* wild type (*n* = 241). Kaplan–Meier curves were plotted using SPSS (version 26) for the *TP53* mutated group and *TP53* wild-type group. Each group was further divided into two sub-groups based on whether it was above or below the median of *BCAR1* expression. A log-rank test was used to compare the survival curves between *BCAR1* high and low gene expression groups. *P* values < 0.05 were considered statistically significant.

### Generation of *TP53*^*–/–*^ MDA-MB-468 clones

*TP53* knockout MDA-MB-468 cells were generated as previously described.^27^ Briefly, MDA-MB-468 cells were transfected with either a gRNA targeting exon 4 of *TP53* (GGCAGCTACGGTTTCCGGTCT) or empty vector together with Cas9-EGFP. Cells were sorted for GFP-positive cells using BD FACSArial II cell sorter (BD Biosciences) and plated sparsely onto tissue cultured plastic plates. Colonies of cells were picked using sterilised cloning cylinders and expanded. Protein analysis was performed to validate the absence of TP53.

### Retroviral vectors and retroviral transduction

To generate retroviruses carrying shRNAs against human *TP53* or *BCAR1*, *TP53* targeting sequence #1 (GGTGAACCTTAGTACCTAA), *TP53* targeting sequence #2 (GTAATCTACTGGGACGGAA), *BCAR1* target sequence #1 (GGATGGAGGACTATGACTA), and *BCAR1* target sequence #2 (GAGTTTGAGAAGACCCAGA) were cloned into a pSuper retro puro (Oligoengine) or pSuper retro hygro vector; the latter was generated by replacing the sequencing encoding puromycin with that encoding hygromycin. Retroviral transduction was performed as previously described.^27^ Briefly, retrovirus vector containing shRNA sequence together with amphotropic retroviral plasmids were co-transfected into HEK 293T cells to generate viral particles. Cells at 50% confluence were infected with retrovirus overnight in the presence of 8 µg/mL polybrene (Sigma Aldrich) and selected with 0.5 µg/mL puromycin or 300 µg/mL hygromycin for 4 days after transduction.

### Small interfering RNA (siRNA) and transfection

Calcium phosphate transfection protocol was performed as described^28^ to introduce control siRNA (D-001810-10-20, ON-TARGETplus Non-targeting Pool, Dharmacon GE Healthcare Bio-Sciences, Lafayette, CO, USA) or siRNA targeting human *TP53* or *BCAR1* at a final concentration of 40 nM into H1299 or A431 cells.

*TP53* targeting sequence forward: GUAAUCUACUGGGACGGAAdTdT

*TP53* targeting sequence reverse: UUCCGUCCCAGUAGAUUACdTdT

*BCAR1* targeting sequence #1 forward: GGAUGGAGGACUAUGACUAdTdT

*BCAR1* targeting sequence #1 reverse: UAGUCAUAGUCCUCCAUCCdAdG

*BCAR1* targeting sequence #2 forward: GAGUUUGAGAAGACCCAGAdTdT

*BCAR1* targeting sequence #2 reverse: UCUGGGUCUUCUCAAACUCdCdT

## Results

### TP53^R273H^ binds to BCAR1 in the nucleus

**Fig. 1:**
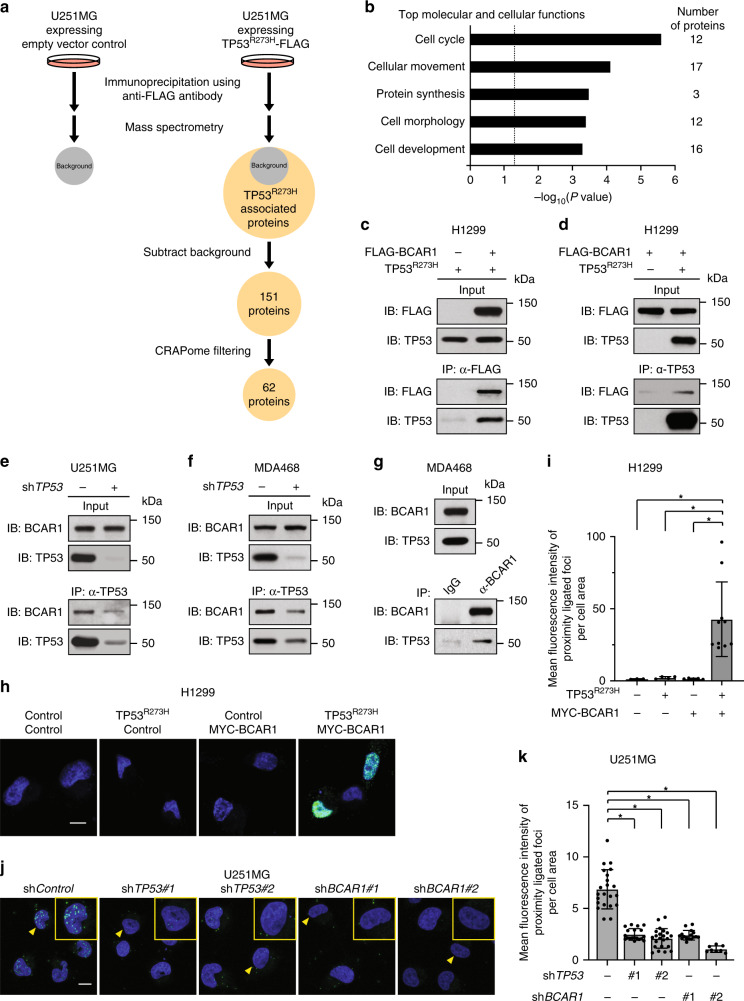
TP53^R273H^ interacts with BCAR1. **a** A schematic workflow of affinity purification coupled to mass spectrometry. Immunoprecipitation with FLAG antibody was performed using U251MG cells (TP53^R273H^-harbouring human glioblastoma) overexpressing TP53^R273H^-FLAG or control vector. Proteins pulled down by FLAG antibody were subjected to mass spectrometry analysis. Proteins identified in cells expressing control vector were considered to bind non-specifically to the antibody (background). **b** Sixty-two proteins identified as candidate interactors were sorted based on their molecular and cellular functions using Ingenuity Pathway Analysis. The top five molecular and cellular functions are shown with the number of proteins under each category indicated. Bars represent the level of significance for the genes associated with the indicated molecular and cellular functions. The *P* value threshold (dotted line) is set at 0.05, indicating a maximum false discovery rate of 5%. **c**, **d** Co-immunoprecipitation of exogenous TP53^R273H^ and FLAG-BCAR1. Lysates of H1299 cells transfected with the indicated plasmids were immunoprecipitated with anti-FLAG antibody (**c**) or with anti-TP53 antibody (**d**), and analysed by Western blot. A FLAG tag-containing vector was used as a negative control for FLAG-BCAR1 in (**c**). **e**–**g** Co-immunoprecipitation of endogenous TP53^R273H^ and BCAR1. Lysates of U251MG (**e**) or MDA-MB-468 (MDA468) cells (**f**) expressing sh*Control* (empty vector control) or sh*TP53(#2)* were immunoprecipitated with the anti-TP53 antibody. Lysates of MDA468 cells (**g**) were immunoprecipitated with anti-BCAR1 antibody or control IgG. Immunoprecipitated proteins were analysed by Western blot. **h** Localisation of the exogenous TP53^R273H^–BCAR1 complexes. Confocal microscopy images of green fluorescence signals obtained from the proximity ligation assay are shown. H1299 cells were transfected with the indicated plasmids. Cells transfected with empty and/or MYC tag-containing plasmids served as negative controls. The nuclei were stained with DAPI. Scale bar represents 10 μm. **i** Quantifications of the proximity ligated foci. The images obtained in (**h**) were used for calculation. Bars represent the mean fluorescence intensity of proximity ligated foci in each cell normalised by cell area ± SD. Three or more cells were used for quantifications and indicated as dots. *p value < 0.05. Statistical analysis of data was done by unpaired Student’s two-sided *t* test. **j** Localisation of the endogenous TP53^R273H^–BCAR1 complexes in U251MG cells expressing sh*Control* (empty vector control). Confocal microscopy images of green fluorescence signals obtained from proximity ligation assay are shown. U251MG cells expressing sh*TP53* or sh*BCAR1* served as negative controls. Fields enclosed by the yellow line show the magnified images of selected cells (arrowhead). Scale bar represents 10 μm. **k** Quantifications of the proximity ligated foci. The images obtained in (**j**) were used for calculation. Bars represent the mean fluorescence intensity of proximity ligated foci in each cell normalised by cell area ± SD. Eight or more cells were used for quantifications and indicated as dots. *P value < 0.05. Statistical analysis of data was done by unpaired Student’s two-sided *t* test.

To uncover a novel mechanism by which mutant TP53 exhibits its gain-of-function properties, we looked for TP53^R273H^ interacting proteins that contribute to cancer cell invasion. R273H mutation is one of the hotspot mutations in *TP53* in human cancers.^8,11,12^ We first conducted a large-scale co-immunoprecipitation with a FLAG antibody following overexpression of TP53^R273H^-FLAG in U251MG cells (TP53^R273H^-harbouring human glioblastoma cell line^34^) (Supplementary Fig. S1). The proteins pulled down by the anti-FLAG antibody were subjected to mass spectrometry (Fig. 1a). After background subtraction, this approach identified 151 potential TP53^R273H^-associated proteins (Supplementary Table 1). Fifty-one out of 151 proteins were reported to bind to TP53. Known TP53^R273H^ binding proteins such as MDM2^35^ and NRDC^36^ were also in our list, validating the reliability of our approach in identifying novel TP53^R273H^ interacting proteins. To further eliminate potential background contaminants from our mass spectrometry, we stringently selected proteins with a low CRAPome value of <15/411. We then stratified the remaining 62 proteins according to their molecular and cellular functions using the Ingenuity Pathway Analysis (IPA). The results show that many of the candidate interactors likely function in the cell cycle or cellular movement (Fig. 1b).

Since we were looking for novel TP53^R273H^-interacting proteins that contribute to cancer cell invasion, we focused on the 17 proteins grouped under the ‘cellular movement’ category (Supplementary Table 2). Using IPA, we generated a functional network of the 17 proteins to connect with the individual cellular functions (Supplementary Fig. S2). The protein that had the highest numbers of the functional connections to the cellular movement was breast cancer anti-estrogen resistance 1 (BCAR1). BCAR1 functions as an adaptor protein downstream of several cell-surface receptors including growth factor receptors and integrins.^37^ Activation of these cell-surface receptors induces phosphorylation of 15 tyrosine motifs (YxxP) of BCAR1 by SRC family kinases (SFKs).^38^ BCAR1 promotes EMT, cell motility, and invasion^37^ and is overexpressed in many human cancers including breast,^39,40^ glioma,^38^ prostate,^41^ ovarian,^42^ and hepatocellular carcinoma.^43^ The increased expression of *BCAR1* in these cancers is frequently associated with advanced stage, metastatic properties, and poor survival rates. Given the well-established importance of BCAR1 in cancer and invasion,^37,44–46^ we chose to focus the rest of our study on the TP53^R273H^−BCAR1 interaction.

To verify our mass spectrometry data, we ectopically expressed TP53^R273H^ and FLAG-tagged BCAR1 in *TP53*-null H1299 cells and performed the co-immunoprecipitation assays. TP53^R273H^ was co-immunoprecipitated with FLAG-BCAR1 (Fig. 1c). The binding was further confirmed by reciprocal co-immunoprecipitation (Fig. 1d). To demonstrate that endogenous TP53^R273H^ and BCAR1 also interact under physiological conditions, we conducted co-immunoprecipitation assay using lysates of U251MG and MDA-MB-468 (TP53^R273H^-harbouring human breast cancer cell line^24^) cells expressing either empty vector control or sh*TP53*. The amount of BCAR1 co-immunoprecipitated by TP53^R273H^ was reduced in both cell lines expressing *TP53* shRNA, corresponding to the reduced amount of TP53^R273H^ pulled down (Fig. 1e, f), confirming their endogenous binding. The endogenous binding was also confirmed reciprocally by co-immunoprecipitating TP53^R273H^ with BCAR1 (Fig. 1g).

To examine the localisation of the TP53^R273H^–BCAR1 complexes, we performed PLAs using H1299 cells ectopically expressing TP53^R273H^ and MYC-tagged BCAR1. Confocal microscopy revealed that exogenous   TP53^R273H^ and MYC-BCAR1 were in close proximity mainly in the nucleus (Fig. 1h, i). Consistent with this, endogenous TP53^R273H^ and BCAR1 were also in close proximity in the nucleus in U251MG cells expressing sh*Control* (empty vector control) (Fig. 1j, k). Together, these results suggest that the binding between TP53^R273H^ and BCAR1 occurs mainly in the nucleus.

### Depletion of TP53^R273H^ and BCAR1 reduces cancer cell invasion to a similar extent as single depletion of TP53^R273H^ or BCAR1

**Fig. 2:**
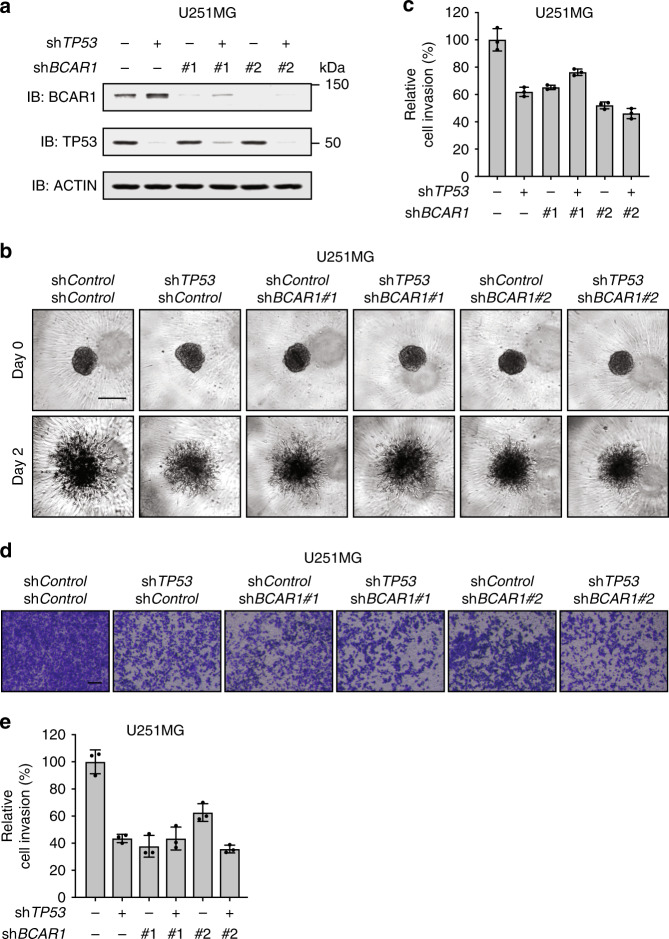
Depletion of TP53^R273H^ and BCAR1 reduces cancer cell invasion to a similar extent as single depletion of TP53^R273H^ or BCAR1. **a** Lysates of U251MG cells transduced with a retrovirus expressing sh*Control* (empty vector control), sh*TP53(#2)*, sh*BCAR1*, or both sh*TP53(#2)* and sh*BCAR1* were analysed by Western blot. **b** Phase-contrast micrographs of 3D spheroid invasion assay using U251MG cells expressing sh*Control* (empty vector control), sh*TP53(#2)*, sh*BCAR1*, or both sh*TP53(#2)* and sh*BCAR1*. Images were taken at the indicated time after spheroids were embedded into Matrigel. Representative images of each group are shown. Scale bar represents 100 μm. **c** Quantification of the area of cells invading into the Matrigel. The images obtained in (**b**) were used for calculation. Bars represent the mean area of cells invading into Matrigel relative to the control (the first bar, set as 100%) ± SD from three independent experiments (*n* = 3). **d** Bright-field micrographs of transwell invasion assay using U251MG cells expressing the indicated shRNA. Representative images of the transwells are shown. Scale bar represents 200 μm. **e** Quantification of the area of cells invading into the Matrigel. The images obtained in (**d**) were used for calculation. Bars represent the mean area of cells invading into Matrigel relative to the control (the first bar, set as 100%) ± SD from three independent experiments (*n* = 3).

It has been reported that TP53^R273H^^19,20,36,47^ as well as BCAR1^37,44–46^ regulate cancer cell invasion. Consistent with these reports, the knockdown of TP53^R273H^ reduced 3D spheroid invasion of U251MG cells (Fig. 2a–c). Knockdown of BCAR1 reduced 3D spheroid invasion to a similar extent as knockdown of TP53^R273H^ (Fig. 2a–c). However, it is not known whether TP53^R273H^ and BCAR1 work cooperatively or separately to promote invasion. If they work cooperatively, then the simultaneous depletion of both proteins should not further reduce 3D spheroid invasion as compared to individual depletion of each protein. Indeed, our results revealed that the invasiveness of U251MG cells was similar when TP53^R273H^ and BCAR1 were depleted simultaneously or individually. We further evaluated cancer cell invasion using a transwell invasion assay. Transwell invasion assay can be done in a shorter time than 3D spheroid invasion assay, thereby minimising the possible influence of cell proliferation on the final readout. Consistent with 3D spheroid invasion assay (Fig. 2b, c), the invasiveness of U251MG cells measured by transwell assay was similar when TP53^R273H^ and BCAR1 were depleted simultaneously or individually (Fig. 2d, e). We confirmed this observation in another cancer cell line, A431 cells (TP53^R273H^-harbouring human epidermoid carcinoma cell line^24^) (Supplementary Fig. S3). Taken together, these results suggest that TP53^R273H^ and BCAR1 may contribute to cancer cell invasion in the same pathway.

### A SRC family kinase inhibitor prevents BCAR1 nuclear import, TP53^R273H^–BCAR1 complex formation, and invasion

**Fig. 3:**
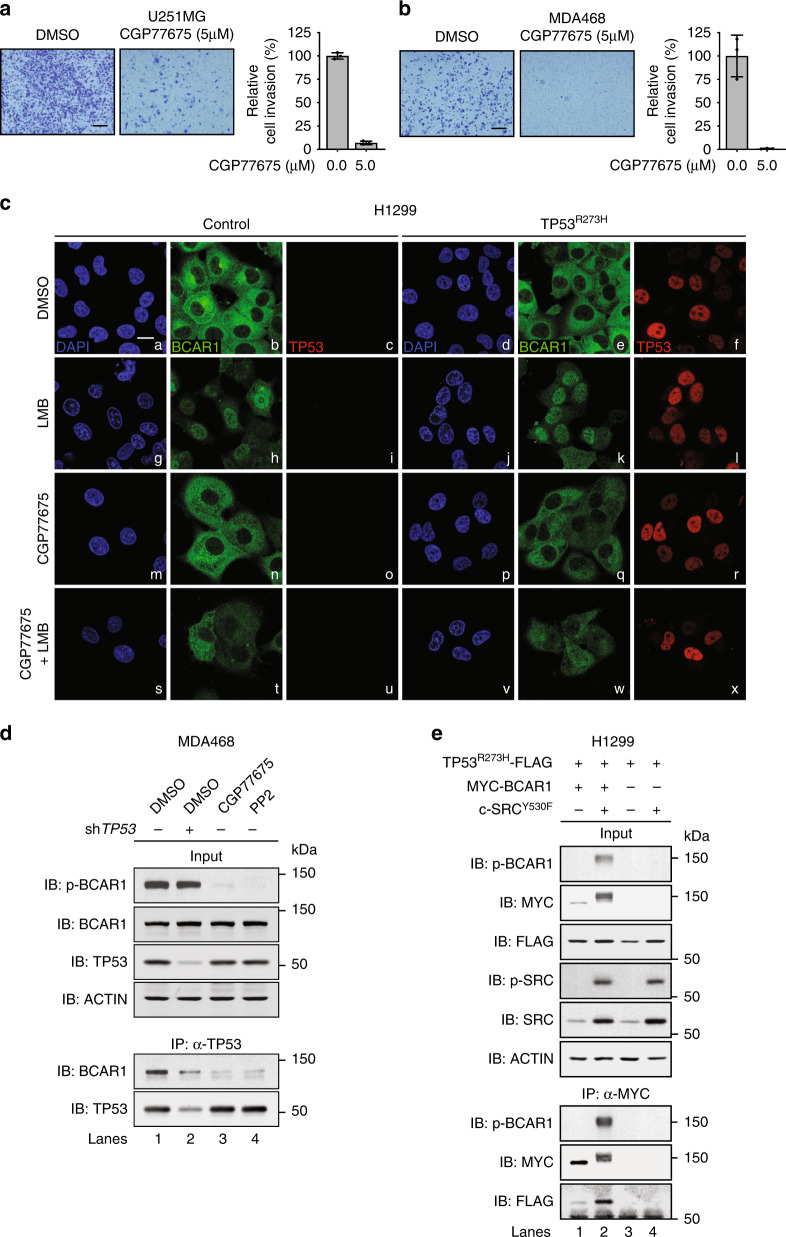
An SRC family kinase inhibitor prevents BCAR1 nuclear import and disrupts the interaction between TP53^R273H^ and BCAR1. **a**, **b** Bright-field micrographs of transwell invasion assay using U251MG (**a**) or MDA-MB-468 (MDA468) cells (**b**) in the absence or presence of CGP77675 (5 µM). Representative images of the transwells are shown. Scale bar represents 200 μm. Quantification of the invading area is shown on the right. Bars represent the mean invading area relative to the DMSO-treated cells (the first bar, set as 100%) ± SD from three independent experiments (*n* = 3). **c** Localisation of BCAR1 and TP53. H1299 cells transfected with *Control* (empty vector control) or *TP53*^*R273H*^ were pre-treated with or without CGP77675 (5 µM) for 9 h, followed by incubation with DMSO, LMB (10 nM), and/or CGP77675 for another 16 h. Cells were then subjected to immunofluorescence staining. Nuclei (blue), endogenous BCAR1 (green) and TP53^R273H^ (red). Scale bar represents 10 μm. **d** Co-immunoprecipitation of BCAR1 with endogenous TP53^R273H^. The cells were treated with DMSO, CGP77675 (5 µM), or PP2 (40 µM) for 16 h. Lysates of sh*Control* (empty vector control)- or sh*TP53(#2)*-expressing MDA-MB-468 (MDA468) cells were immunoprecipitated with anti-TP53 antibody and analysed by Western blot. **e** Co-immunoprecipitation of exogenous TP53^R273H^-FLAG with MYC-BCAR1 in the presence or absence of c-SRC^Y530F^. Lysates of H1299 cells transfected with the indicated plasmids were immunoprecipitated with anti-MYC antibody and analysed by Western blot. An empty vector or MYC tag-containing plasmid was used as a negative control.

SFKs phosphorylate BCAR1 and promote cancer cell invasion.^37,48^ To evaluate whether SFKs are involved in the TP53^R273H^−BCAR1 interaction and cancer cell invasion, we first examined whether inhibition of SFKs prevents the invasiveness of U251MG and MDA-MB-468 cells. Consistent with previous findings,^49^ treatment with SFKs inhibitor CGP77675 abolished the invasion of U251MG and MDA-MB-468 cells (Fig. 3a, b).

We next examined the effects of SFKs inhibition on the spatial localisation of TP53^R273H^ and BCAR1 in cells by immunofluorescence staining. The specificity of the BCAR1 antibody was confirmed by the staining of H1299 cells expressing control siRNA or siRNA targeting *BCAR1* (Supplementary Fig. S4). The majority of endogenous BCAR1 localised in the cytoplasm in *TP53*-null H1299 cells (Fig. 3c, panel b). Cytoplasmic localisation of BCAR1 was not affected by ectopic expression of TP53^R273H^ (Fig. 3c, panel e), which primarily localised in the nucleus (Fig. 3c, panel f). This indicates that TP53^R273H^ does not promote BCAR1 nuclear translocation. Consistent with this result, endogenous BCAR1 also localised in the cytoplasm regardless of the presence or absence of endogenous TP53^R273H^ expression (Supplementary Fig. S5).

BCAR1 has been reported to shuttle between the nucleus and the cytoplasm.^50–52^ We speculated that BCAR1 nuclear export is stronger than nuclear import since we observed BCAR1 predominantly in the cytoplasm. Indeed, when the cells were treated with leptomycin B (LMB), an exportin inhibitor to inhibit protein export activity, BCAR1 was accumulated in the nucleus regardless of the TP53^R273H^ expression (Fig. 3c, panels h, k). LMB treatment also slightly increased TP53^R273H^−BCAR1 binding (Supplementary Fig. S6). BCAR1 contains 15 tyrosine residues within its substrate domain, which are phosphorylated mainly by SFKs.^37,38^ One study has revealed that phosphorylation of BCAR1 is required for nuclear import,^53^ while another demonstrated that BCAR1 could be imported into the nucleus in the absence of phosphorylation.^50^ Since the role for phosphorylation in nuclear import of BCAR1 remains controversial, we examined whether SFKs-mediated BCAR1 phosphorylation is required for nuclear import of BCAR1 in our study. However, it was difficult to assess whether blocking BCAR1 phosphorylation with a SFKs inhibitor CGP77675 attenuates nuclear import since BCAR1 was already predominantly localised in the cytoplasm due to the strong nuclear export activity, regardless of treatment with CGP77675 (Fig. 3c, panels b, n). Therefore, we treated the cells with CGP77675 in the presence of LMB to make it easier to observe the effect of CGP77675 on BCAR1 nuclear transport. Nuclear accumulation of BCAR1 by LMB (Fig. 3c, panel h) was attenuated when cells were treated with both CGP77675 and LMB (Fig. 3c, panel t). This was also observed in cells expressing exogenous TP53^R273H^ (Fig. 3c, panels k, w). These results suggest that SFKs-mediated phosphorylation of BCAR1 is required for nuclear translocation regardless of TP53^R273H^ expression.

The amount of phosphorylated BCAR1 remained unchanged upon depletion of TP53^R273H^ (Fig. 3d, lanes 1–2). These data led us to speculate that, regardless of TP53^R273H^ expression, once BCAR1 gets phosphorylated, a subset of BCAR1 translocates into the nucleus where it interacts with TP53^R273H^. Hence, we expected that the interaction between TP53^R273H^ and BCAR1 would be abolished if BCAR1 phosphorylation was inhibited by SFKs inhibitors, either CGP77675 or PP2.^54^ Indeed, the amount of endogenous BCAR1 that was co-immunoprecipitated with endogenous TP53^R273H^ was markedly reduced in CGP77675- or PP2-treated cells as compared to DMSO-treated cells (Fig. 3d, lanes 1, 3–4). Furthermore, the expression of constitutively activated c-SRC (c-SRC^Y530F^) enhanced the phosphorylation of BCAR1 remarkably and increased the amount of TP53^R273H^ that was co-immunoprecipitated with BCAR1 (Fig. 3e, lanes 1–2).

Taken together, these data indicate that TP53^R273H^ is not involved in SFKs-mediated phosphorylation of BCAR1 and translocation of phosphorylated BCAR1 into the nucleus, but rather suggest that TP53^R273H^ forms a complex with the nuclear-imported BCAR1 that is phosphorylated by SFKs.

### BCAR1 interacts with the DNA-binding domain region 102–207 of TP53^R273H^

**Fig. 4:**
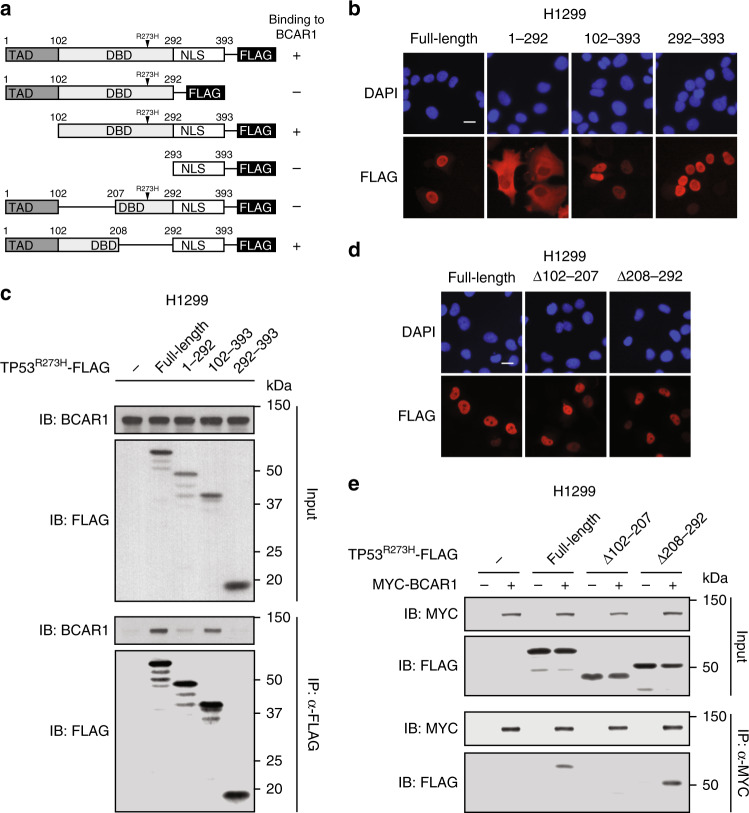
BCAR1 interacts with the DNA-binding domain region 102–207 of TP53^R273H^. **a** Schematic of full-length and deletion mutants of TP53^R273H^-FLAG constructs and their binding status to BCAR1. Transactivation domain (TAD); DNA-binding domain (DBD); Nuclear localisation domain (NLS). **b** Localisation of full-length or deletion mutants of TP53^R273H^-FLAG. Fluorescence images of nuclei (blue) or TP53^R273H^-FLAG (red) are shown. H1299 cells were transfected with the indicated plasmids. The nuclei were stained with DAPI. Scale bar represents 20 μm. **c** Co-immunoprecipitation of endogenous BCAR1 with full-length or deletion mutants of TP53^R273H^-FLAG. Lysates of H1299 cells expressing the indicated constructs were immunoprecipitated with anti-FLAG antibody and analysed by Western blot. A FLAG tag-containing plasmid was used as a negative control. **d** Localisation of full-length or deletion mutants of TP53^R273H^-FLAG. Fluorescence images of nuclei (blue) or TP53^R273H^-FLAG (red) are shown. H1299 cells were transfected with the indicated plasmids. The nuclei were stained with DAPI. Scale bar represents 20 μm. **e** Co-immunoprecipitation of deletion mutants of TP53^R273H^-FLAG with exogenous MYC-BCAR1. Lysates of H1299 cells expressing the indicated constructs were immunoprecipitated with anti-MYC antibody and analysed by Western blot. A FLAG or MYC tag-containing plasmid was used as a negative control.

To evaluate whether the interaction between TP53^R273H^ and BCAR1 is crucial for promoting cancer cell invasion, we first generated FLAG-tagged full-length and deletion mutants of TP53^R273H^ to identify which region is important for BCAR1 interaction (Fig. 4a). Our immunofluorescence staining revealed that two truncation mutants, TP53^R273H^ (102–393) and TP53^R273H^ (293–393), localised in the nucleus similar to TP53^R273H^ full-length (Fig. 4b). Despite similar nuclear localisation patterns, full-length TP53^R273H^ and TP53^R273H^ (102–393) but not TP53^R273H^ (293–393) was immunoprecipitated with endogenous BCAR1 (Fig. 4c), suggesting that BCAR1 binds to the region corresponding to amino acids 102–292 of TP53^R273H^. On the other hand, TP53^R273H^ (1–292) did not bind efficiently to BCAR1 even though it contains the 102–292 region (Fig. 4c). We speculate that this is because TP53^R273H^ (1–292) localised to the cytoplasm due to the lack of the nuclear localisation signal (NLS) of TP53^R273H^ located in the 293–393 region (Fig. 4b). The reduction in the interaction between cytoplasmic TP53^R273H^ (1–292) and BCAR1 further supports our earlier findings that the interaction takes place mainly in the nucleus (Fig. 1h, j). Consistent with these results, PLA revealed that the cells co-expressing TP53^R273H^ (1–292) and MYC-BCAR1 have low fluorescence signals in the cytoplasm similar to the low signals found in the cytoplasm of some cells co-expressing TP53^R273H^ full-length and MYC-BCAR1 (Fig. 1h and Supplementary Fig. S7). Taken together, these results suggest that BCAR1 binds to the 102–292 region of TP53^R273H^ predominantly in the nucleus.

To further narrow down the binding region within 102–292 of TP53^R273H^, we generated TP53^R273H^Δ102–207 and TP53^R273H^ Δ208–292 deletion mutants. Immunofluorescence staining confirmed that both TP53^R273H^Δ102–207 and TP53^R273H^Δ208–292 localised in the nucleus (Fig. 4d). We performed co-immunoprecipitation assays and found that MYC-BCAR1 bound to TP53^R273H^ full-length and TP53^R273H^Δ208–292, but not TP53^R273H^Δ102–207 (Fig. 4e). These data suggest that BCAR1 binds to TP53^R273H^ within the region corresponding to amino acids 102–207.

### Disruption of the TP53^R273H^−BCAR1 interaction reduces invasion

**Fig. 5:**
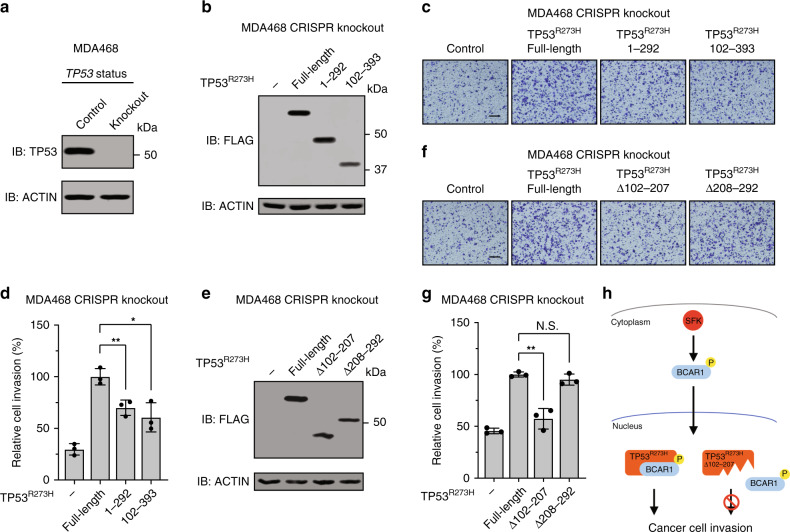
Disruption of the TP53^R273H^−BCAR1 interaction reduces invasion. **a** Lysates of MDA-MB-468 (MDA468) CRISPR control or knockout cells were analysed by Western blot. **b** Lysates of MDA468 *TP53*^*–/–*^ cells expressing the indicated constructs were analysed by Western blot. **c** Bright-field micrographs of transwell invasion assay using MDA468 *TP53*^*–/–*^ cells expressing the indicated plasmids. Representative images of the transwells are shown. Scale bar represents 200 μm. **d** Quantification of the invading area. The images obtained in (**c**) were used for calculation. Bars represent the mean area of cells invading Matrigel relative to full-length (the second bar, set as 100%) ± SD from three independent experiments (*n* = 3). *P < 0.05. **P value < 0.01. Statistical analysis of data was done by unpaired Student’s two-sided *t* test. **e** Lysates of MDA468 *TP53*^*–/–*^ cells expressing the indicated constructs were analysed by Western blot. **f** Bright-field micrographs of transwell invasion assay using MDA468 *TP53*^*–/–*^ cells expressing the indicated plasmids. Representative images of the transwells are shown. Scale bar represents 200 μm. **g** Quantification of the invading area. The images obtained in (**f**) were used for calculation. Bars represent the mean area of cells invading Matrigel relative to full-length (the second bar, set as 100%) ± SD from three independent experiments (*n* = 3). **P value < 0.01. NS = not significant. Statistical analysis of data was done by unpaired Student’s two-sided *t* test. **h** Schematic model of the TP53^R273H^–BCAR1 complex-mediated cancer cell invasion. SRC family kinases (SFKs) promote BCAR1 phosphorylation and nuclear translocation. Full-length TP53^R273H^, but not the BCAR1 binding-deficient mutant (TP53^R273H^Δ102–207), enhances cancer cell invasion by binding to BCAR1 in the nucleus. P stands for phosphorylation.

To test whether the interaction between TP53^R273H^ and BCAR1 is essential for cancer cell invasion, we evaluated the invasiveness of MDA-MB-468 *TP53*^*–/–*^ cells expressing TP53^R273H^ full-length or deletion mutants by transwell invasion assay. *TP53*^*R273H*^ knockout MDA-MB-468 human breast cancer cells were generated using the CRISPR/Cas9 system (Fig. 5a), and exogenous TP53^R273H^ full-length or deletion mutants were introduced by transient transfection (Fig. 5b). Consistent with the reported invasive phenotype of TP53^R273H^,^19,20,36,47^ TP53^R273H^ full-length promoted transwell invasion compared to the empty vector control (Fig. 5c, d). As we expected, BCAR1 binding-deficient-TP53^R273H^ (1–292) that does not localise in the nucleus did not promote transwell invasion as strongly as TP53^R273H^ full-length (Fig. 5c, d). However, TP53^R273H^ (102–393), which binds to BCAR1, also did not promote transwell invasion to a similar extent as TP53^R273H^ full-length (Fig. 5c, d). This could be due to the lack of the transactivation domain of TP53^R273H^, which is essential for some of the mutant TP53 gain-of-function effects.^18,55–57^ Therefore, we decided to use the deletion mutants that retain the transactivation domain for further assessment (Fig. 5e). TP53^R273H^Δ208–292, which binds to BCAR1, promoted transwell invasion to a similar extent as TP53^R273H^ full-length (Fig. 5f, g). By contrast, TP53^R273H^Δ102–207, which does not bind to BCAR1, did not promote transwell invasion (Fig. 5f, g). Together, these results suggest that the interaction between TP53^R273H^ and BCAR1 in the nucleus (in the presence of an intact transactivation domain of TP53^R273H^) is responsible for promoting cancer cell invasion (Fig. 5h).

### Higher *BCAR1* expression with *TP53* mutations confers poorer overall survival in cancer patients

We next examined whether there is a positive correlation between TP53^R273H^ and BCAR1 expression. The depletion of TP53^R273H^ expression did not reduce the BCAR1 protein amounts in U251MG (Fig. 1e), MDA-MB-468 (Fig. 1f), and A431 cells (Supplementary Fig. S3). Conversely, overexpression of TP53^R273H^ did not increase the endogenous BCAR1 protein amounts in H1299 cells (Fig. 4c). Furthermore, there was no clear positive correlation in the expression of TP53^R273H^ and BCAR1 between *TP53*-null H1299 cancer cells and multiple TP53^R273H^-harbouring cancer cells (Supplementary Fig. S8). Likewise, the depletion of BCAR1 did not reduce TP53^R273H^ expression in U251MG (Fig. 2a) and A431 cells (Supplementary Fig. S3). Together, these results indicate that there is no positive correlation between TP53^R273H^ and BCAR1 expression.

**Fig. 6:**
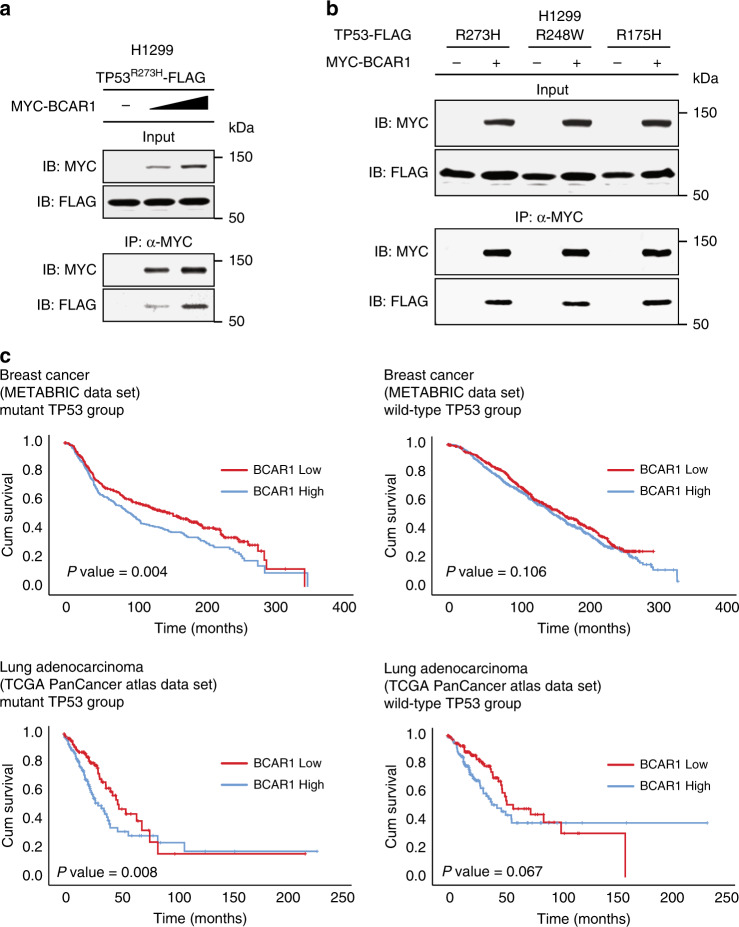
Higher *BCAR1* expression with *TP53* mutations confers poorer overall survival in cancer patients. **a** Co-Immunoprecipitation of exogenous TP53^R273H^-FLAG with MYC-BCAR1. H1299 cells were transfected with TP53^R273H^-FLAG together with the increasing amounts of MYC-BCAR1 plasmids. Lysates were immunoprecipitated with anti-MYC antibody and analysed by Western blot. An MYC tag-containing plasmid was used as a negative control. **b** Co-immunoprecipitation of exogenous FLAG-tagged various mutant TP53 with MYC-BCAR1. Lysates of H1299 cells transfected with the indicated plasmids were immunoprecipitated with anti-MYC antibody and analysed by Western blot. An MYC tag-containing plasmid was used as a negative control. **c** Kaplan−Meier plots using a dataset from METABRIC for breast cancer and a dataset from TCGA PanCancer Atlas for lung adenocarcinoma. The datasets were stratified according to the *TP53* status (wild type or mutant) and *BCAR1* expression levels; higher (high) or lower (low) than the median. Censored cases are depicted.

As higher BCAR1 expression is associated with poorer prognosis,^37,39,45,58^ we next examined whether the TP53^R273H^–BCAR1 binding is affected by the amount of BCAR1 protein expression. We performed co-immunoprecipitation assays using the cells expressing the increasing amounts of BCAR1. We demonstrated that more TP53^R273H^ was co-immunoprecipitated with the increasing amount of MYC-BCAR1 (Fig. 6a). This result led us to speculate that higher expression of BCAR1 in the presence of TP53^R273H^ enhances the TP53^R273H^–BCAR1 protein binding, thereby adversely affecting the survival outcome of cancer patients. To extend our speculation to other hotspot mutants of TP53, we also showed that BCAR1 interacted with TP53^R248W^ and TP53^R175H^ to a similar extent as TP53^R273H^ (Fig. 6b). This result indicates that BCAR1 can bind to other mutant TP53, and its binding is not restricted to TP53^R273H^ specifically. We then retrieved the clinical data from cBioportal database for breast cancer (METABRIC dataset) and for lung adenocarcinoma (TCGA PanCancer Atlas dataset) and plotted the Kaplan−Meier curves for each *TP53* status (wild type or mutant group) (Fig. 6c). All the *TP53* mutations, including *TP53*^*R273H*^, were classified into the mutant group. We further divided each group into two sub-groups based on whether it was above or below the median of *BCAR1* expression. The Kaplan−Meier curves showed that, among the patients with *TP53* mutations, those with high *BCAR1* expression had significantly shorter overall survival than those with low *BCAR1* expression. In wild-type *TP53* patients, on the other hand, there was no significant difference in overall survival between high and low *BCAR1* expression. These data suggest that the TP53^R273H^ and BCAR1 may work together to adversely affect overall survival in cancer patients.

## Discussion

Mutant TP53 interacts with multiple proteins to exert gain-of-function properties for tumour progression. We have discovered a novel interaction between TP53^R273H^ and BCAR1 through the DNA-binding domain of TP53^R273H^ and propose the importance of this interaction in promoting cancer cell invasion.

Many studies have shown that mutant forms of TP53 bind to several transcription factors, including SP1, ETS1, ETS2, VDR, SREBP-2, and NF-Y, altering expression of their target genes to produce gain-of-function properties.^8,21,23^ We showed that TP53^R273H^ interacts with BCAR1 in the nucleus and that the TP53 transactivation domain and the nuclear localisation of both TP53^R273H^ and BCAR1 are required to promote cancer cell invasion. These results suggest that the TP53^R273H^–BCAR1 complex may contribute to transcriptional regulation. However, BCAR1 is not a transcription factor, and it does not have any DNA-binding domain or transactivation domain. Therefore, BCAR1 may act as a bridge protein to recruit TP53^R273H^ to certain transcription factors. For example, TOPBP1 is required for the interaction between TP53^R273C^ and the transcription factor NF-Y to enhance NF-Y target gene expression.^59^ BCAR1 was reported to interact with zinc finger protein ZNF384, which is a transcription factor that regulates extracellular matrix remodelling.^37,45,48,60,61^ However, we could not detect ZNF384 in the proteins pulled down by TP53^R273H^ in our mass spectrometry screen, although we isolated several other transcription factors including members of the zinc finger protein family. Further work is required to uncover the transcription factors involved in the TP53^R273H^−BCAR1 complex-mediated transcriptional regulation.

Most TP53 mutations are missense mutations that result in amino acid substitutions within the DNA-binding domain of TP53.^8^ These mutations are widely thought to result in loss of TP53 transcriptional function due to loss of its DNA-binding ability. Although some forms of TP53 with a mutated DNA-binding domain can still bind to TP53-responsive elements derived from *CDKN1A*, *BAX* and *IGFBP3* promoters,^62^ TP53^R273H^ used in our study does not directly bind to the responsive elements of wild-type TP53.^63^ However, we cannot completely eliminate the possibility that the TP53^R273H^−BCAR1 complexes directly bind to promoters since it has been shown that mutant p53 does bind to certain DNA structural motifs.^8^ Further studies are needed to address whether the TP53^R273H^−BCAR1 complex itself can access promoter regions of pro-invasive genes to enhance the invasiveness phenotype.

Mutant TP53 is reported to enhance cancer cell invasion by promoting RAB11FIP1/Rab coupling protein (RCP)-mediated integrin recycling via the endosomal pathway.^19^ It has not been examined whether BCAR1 is involved in this process. BCAR1 is an adaptor protein that is often recruited to integrins as part of the focal adhesion assembly, where BCAR1 can be phosphorylated by SFKs.^37^ A recent study has demonstrated that SFKs co-localise with endocytosed integrin and that treatment with SFKs inhibitors interrupts the integrin recycling process.^64^ Although we do not exclude the possibility that cytoplasmic BCAR1 and SFKs are involved in mutant TP53-dependent integrin recycling as an essential component for focal adhesion, we have found a previously unappreciated BCAR1 function in the nucleus.

In contrast to mutant TP53, wild-type TP53 suppresses SRC-induced invadopodia, actin-rich structures that promote cancer cell invasion, through the induction of tumour suppressor PTEN (phosphatase and tensin homologue).^65^ PTEN inactivates SRC kinase by dephosphorylating its tyrosine 418 residue.^65^ It is, therefore, possible that the mutation in TP53 results in the reduction of PTEN expression, leading to SRC activation, which promotes BCAR1 phosphorylation, nuclear import of BCAR1, and TP53^R273H^−BCAR1 complex formation, thereby increasing the invasive phenotype.

Recent evidence suggests that not all hotspot mutants of TP53, including those with mutations at residues R175, R248, and R273, have the same gain-of-function phenotypes, and each underlying mechanism may differ.^13,66^ Although we showed that TP53^R175H^, TP53^R248W^, and TP53^R273H^ interact with BCAR1, we have not determined whether TP53^R175H^ and TP53^R248W^ bind to BCAR1 through the same 102–207 region of TP53^R273H^. Since TP53^R175H^ is structurally different from TP53^R273H^ and TP53^R248W^,^67^ TP53^R175H^ might bind to BCAR1 differently. While the patients with mutant *TP53* and high *BCAR1* expression have shorter overall survival than the patients with low *BCAR1* expression, we are unclear how much the mutant TP53−BCAR1 complex-mediated cancer cell invasion affects the reduction in overall survival. Besides the cancer cell invasion, different TP53 mutants may exert a variety of gain-of-function including drug resistance.^24,68^ Drug resistance is an important factor that influences the overall survival of patients.^69^ BCAR1 is also known to promote drug resistance.^37,45^ Therefore, the underlying mechanisms of how these interactions affect patient survival remain to be investigated.

High *BCAR1* expression was not correlated with poorer overall survival among breast cancer and lung adenocarcinoma patients with wild-type *TP53*. This suggests, at least in part, high *BCAR1* expression itself is insufficient to drive breast cancer progression without the cooperation of mutant TP53. However, BCAR1 also has other binding partners which contribute to tumour progression.^37,70^ These binding proteins can be differentially expressed in different cancers and may affect patient survival to various degrees. Therefore, depending on the types of cancers, high *BCAR1* expression could adversely affect patient survival even without mutant TP53. That might be one of the reasons why we observed the different trends in survival curves between breast cancer and lung adenocarcinoma with wild-type *TP53* and high/low *BCAR1* expression, although there was no statistical significance in both cancer patients. There may be many other factors that affect the survival curves, such as various tumour-suppressive activities of wild-type TP53 in each tissue,^1^ which warrant further investigation.

Our results showed that TP53^R273H^ did not promote nuclear translocation of BCAR1. Instead, the TP53^R273H^−BCAR1 complex formation is dependent on SFKs-mediated BCAR1 nuclear localisation. This may be a regulatory mechanism for cancer cells to avoid constitutive and excessive induction of pro-invasive genes by the TP53^R273H^−BCAR1 complex. The TP53^R273H^−BCAR1 complex may induce gene expression only when SFKs are activated by upstream signals triggered by either binding between the extracellular matrix and integrin receptors or binding between growth factors and cell-surface receptors such as receptor tyrosine kinases.^37^ The development of drugs that block BCAR1 phosphorylation and translocation into the nucleus, interfere with the TP53^R273H^−BCAR1 interaction, or degrade TP53^R273H^, could be effective strategies to inhibit the TP53^R273H^−BCAR1-mediated invasion in cancer. Supporting this idea, drugs designed to degrade mutant TP53, including 17-AAG (HSP90 inhibitor) and SAHA (histone deacetylase inhibitor), effectively reduce the invasiveness of grafted tumours of MDA-MB-468 cells carrying the TP53^R273H^ mutation in mouse xenograft models.^71^ Taken together, the results presented here can potentially be used to explore novel therapeutic options for cancer patients carrying mutant TP53.

## Supplementary information

Supplementary Figures

Supplementary Tables

## Data Availability

The data and materials reported in this article are available by the corresponding author upon reasonable request. Clinical datasets are available from public database described in the “Methods” section.
