# Supplementary material for: Mutant TP53 interacts with BCAR1 to contribute to cancer cell invasion
Source: Br J Cancer. 2020 Nov 4;124(1):299–312. doi: 10.1038/s41416-020-01124-9 (PMC7782524; doi:10.1038/s41416-020-01124-9)
Supplement: Supplementary file 1 — Supplementary Figures [file 41416_2020_1124_MOESM1_ESM.pdf]

Mutant TP53 interacts with BCAR1 to contribute to cancer cell invasion
Running title: *$TP53^{R273H}$-BCAR1 complex promotes cancer invasion*

SUPPLEMENTARY INFORMATION

Supplementary Figure S1. Western blot analysis of $TP53^{R273H}$-FLAG in U251MG cells.

Supplementary Figure S2. Functional network of the 17 molecules identified under the cellular movement category.

Supplementary Figure S3. Transwell invasion assay using A431 cells.

Supplementary Figure S4. BCAR1 immunofluorescence staining.

Supplementary Figure S5. BCAR1 and TP53 immunofluorescence staining.

Supplementary Figure S6. Co-immunoprecipitation of BCAR1 and $TP53^{R273H}$ in the presence or absence of leptomycin B treatment.

Supplementary Figure S7. Proximity ligation Assay for $TP53^{R273H}$ and BCAR1.

Supplementary Figure S8. Western blot analysis of $TP53^{R273H}$ and BCAR1.

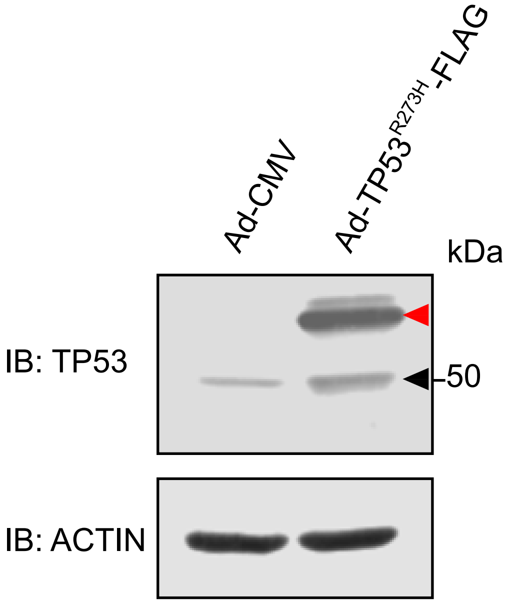

Supplementary Figure S1. Western blot analysis of TP53^{R273H}-FLAG in U251MG cells.
Lysates of U251MG cells transduced with Ad-CMV (empty vector control) or Ad-TP53^{R273H}-FLAG adenovirus were analysed by Western blot using an anti-TP53 antibody (DO-1). Red arrowhead indicates TP53^{R273H} -FLAG. Black arrowhead indicates endogenous TP53^{R273H}.

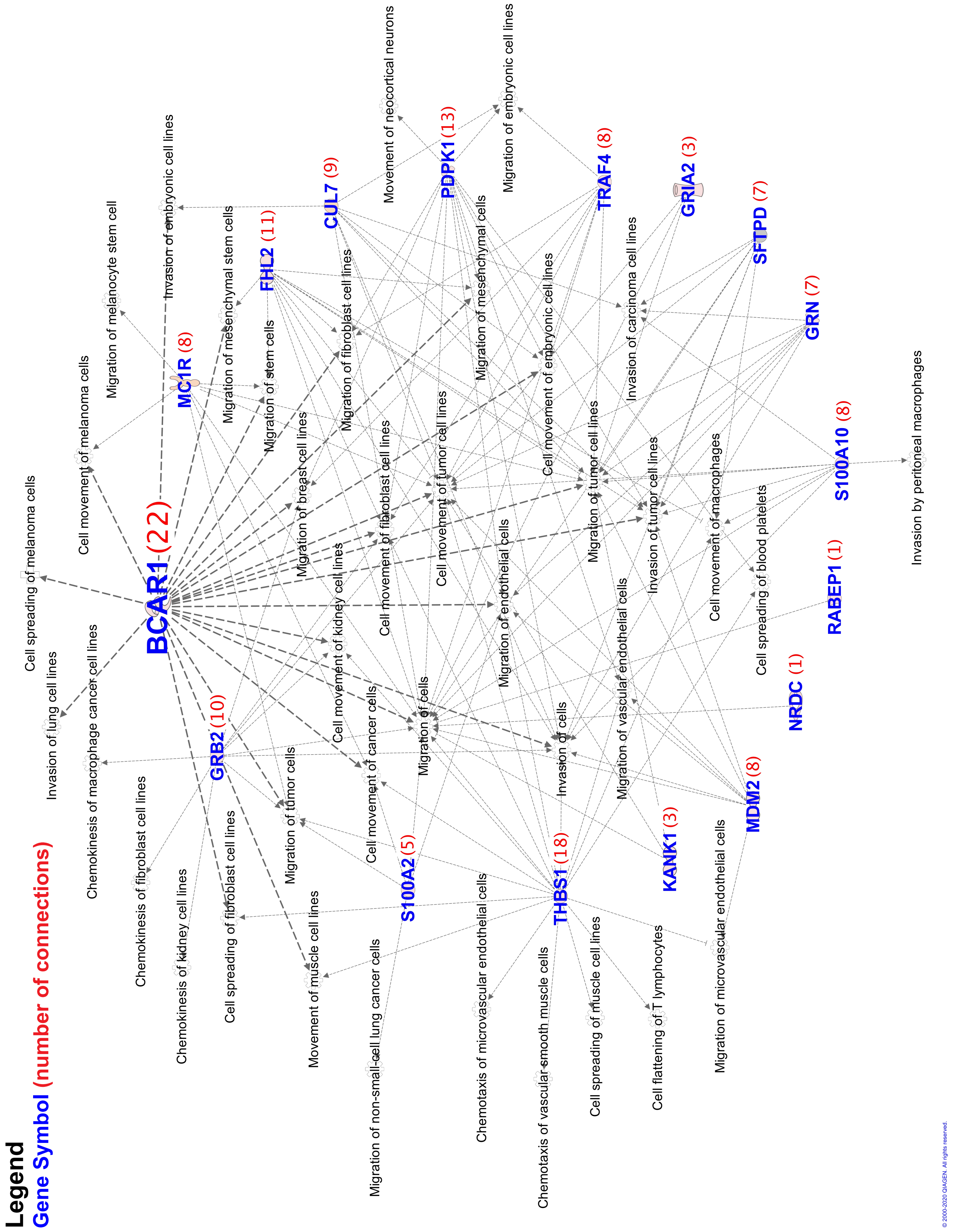

Supplementary Figure S2. Functional network of the 17 molecules identified under the cellular movement category.

The 17 molecules identified under the cellular movement category were used to generate the functional network using Ingenuity Pathway Analysis (IPA). The numbers in red represent the number of functions that each protein is involved in.

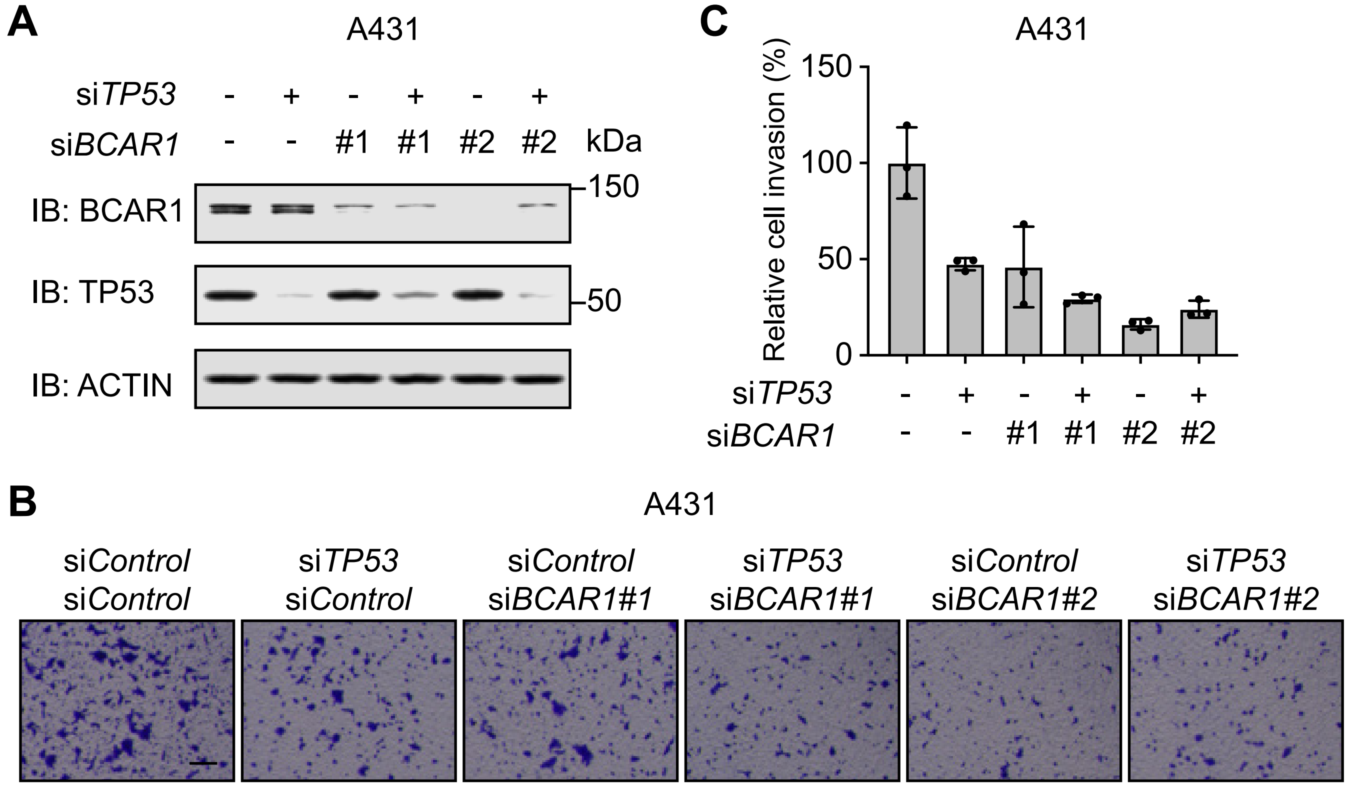

Supplementary Figure S3. Transwell invasion assay using A431 cells.
(A) Lysates of A431 cells transfected with the indicated siRNAs were analysed by Western blot.
(B) Bright-field micrographs of transwell invasion assay using A431 cells transfected with the indicated siRNAs. Representative images of the transwells are shown. Scale bar represents 200 μm.
(C) Quantification of the area of cells invading into the Matrigel. The images obtained in (B) were used for calculation. Bars represent the mean area of cells invading Matrigel relative to the control (the first bar, set as 100%) ± SD from three independent experiments (n=3).

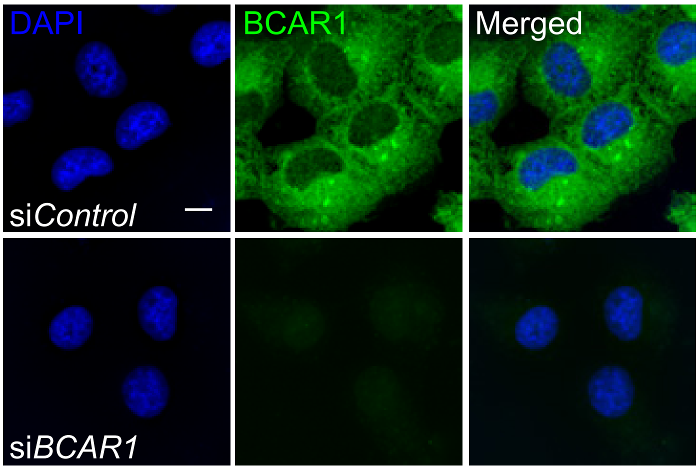

Supplementary Figure S4. BCAR1 immunofluorescence staining.

H1299 cells transfected with either control siRNA (*siControl*) or siRNA targeting BCAR1 (*siBCAR1*) were subjected to immunofluorescence staining. Nuclei were stained with DAPI. Nucleus is in blue and endogenous BCAR1 is in green. Scale bar represents 10 μm.

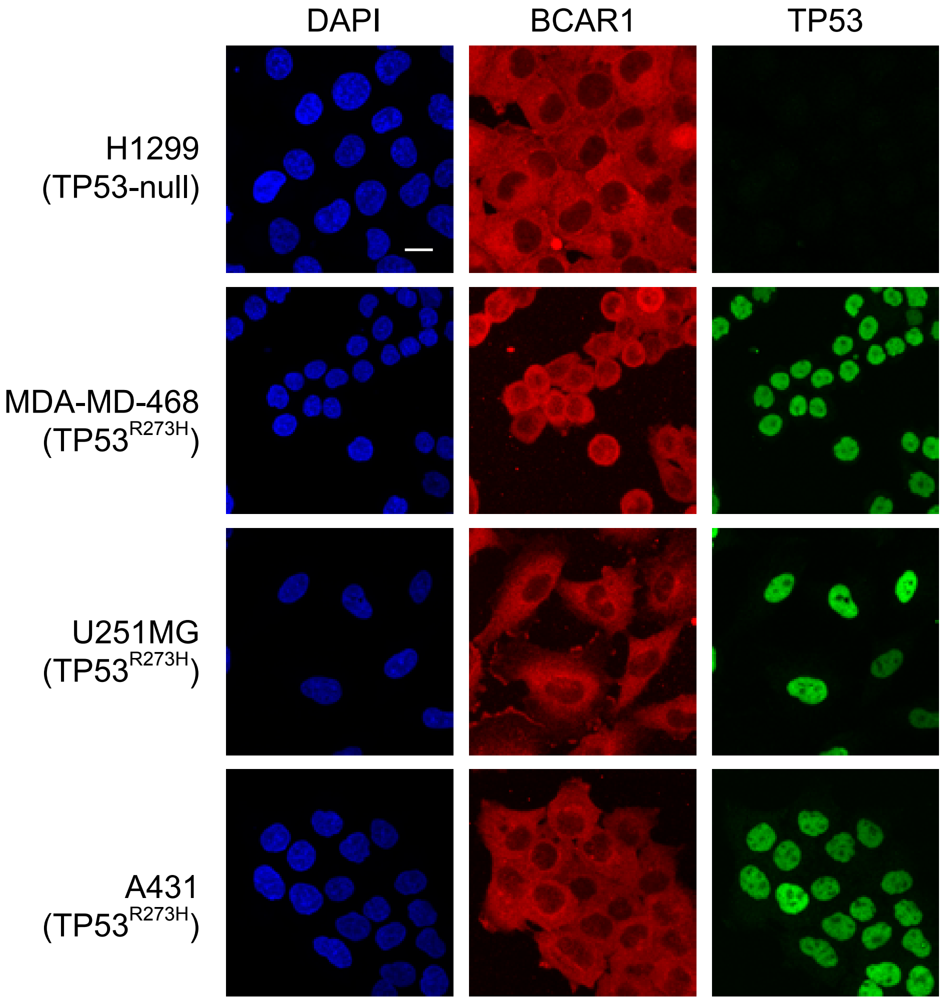

Supplementary Figure S5. BCAR1 and TP53 immunofluorescence staining.

Cells were stained for the nucleus (DAPI, blue), BCAR1 (anti-BCAR1 antibody, red), and TP53 (anti-TP53 antibody, green). Scale bar represents 10 μm.

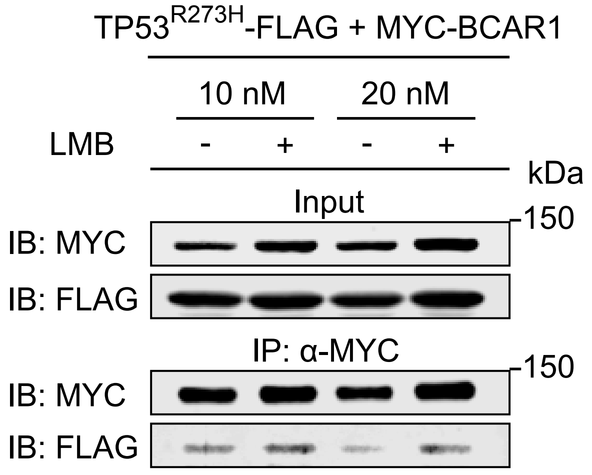

Supplementary Figure S6. Co-immunoprecipitation of BCAR1 and $TP53^{R273H}$ in the presence or absence of leptomycin B treatment.

H1299 cells expressing the indicated constructs were treated with or without leptomycin B (LMB) for 16 hours. Lysates were immunoprecipitated with an anti-MYC antibody and analysed by Western blot using the indicated antibodies.

A

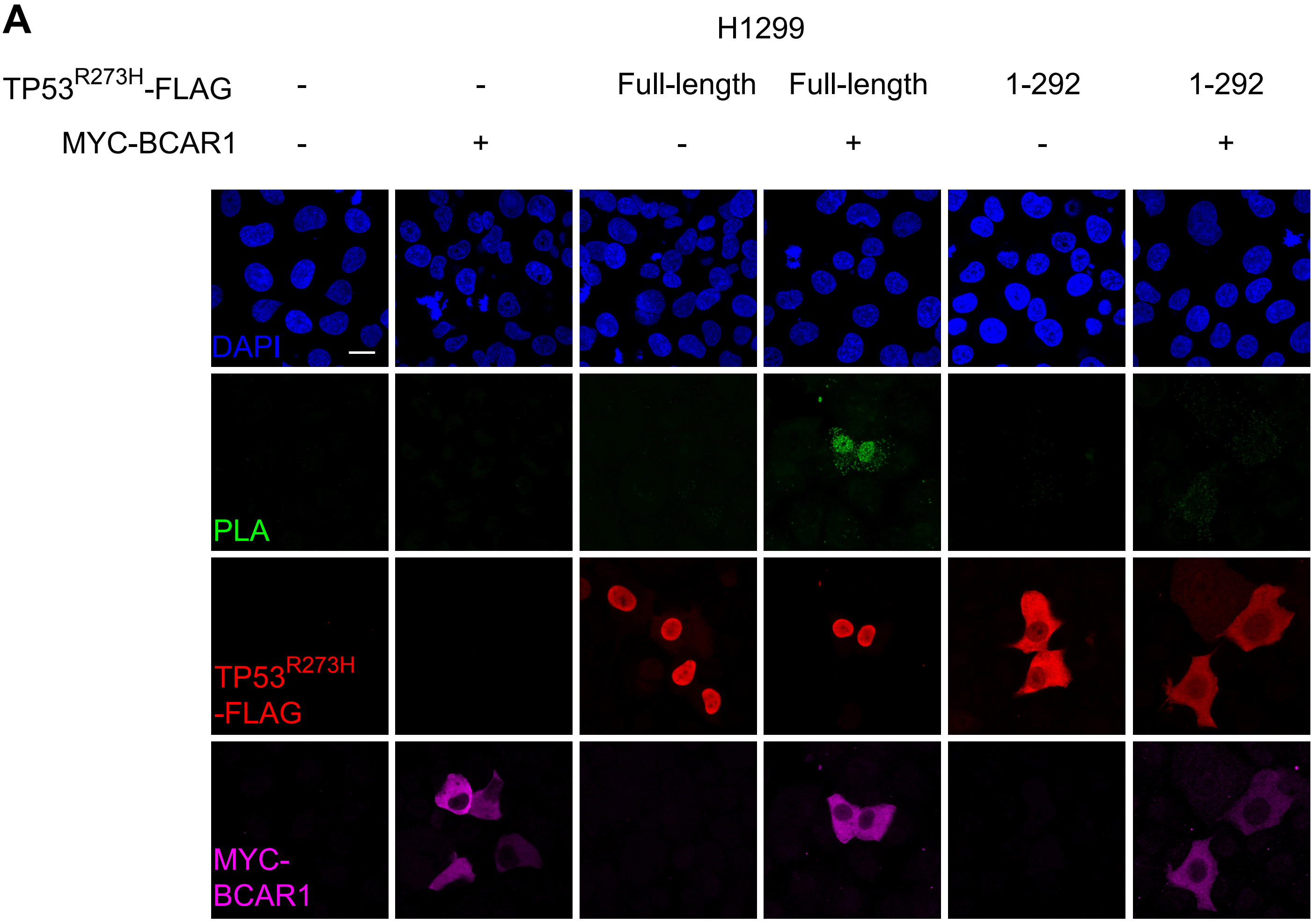

B

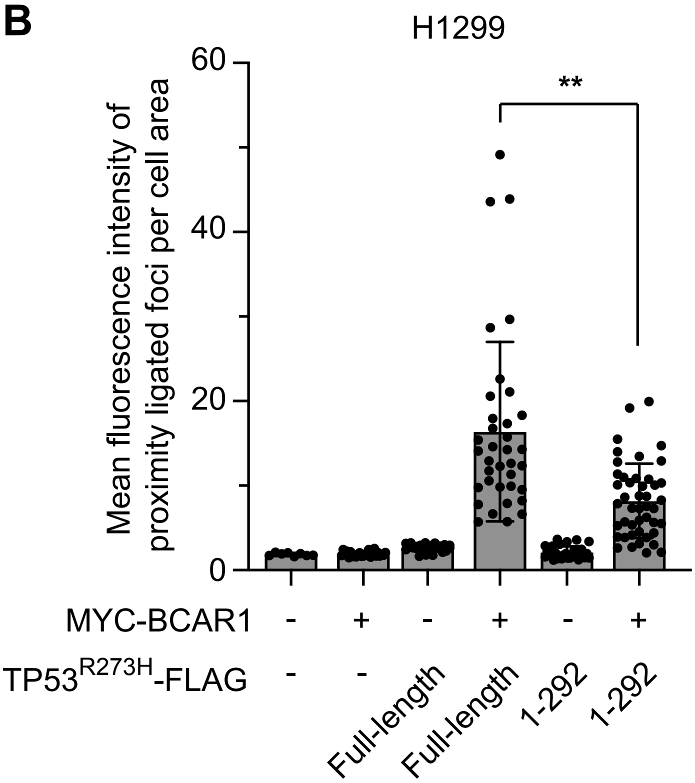

Supplementary Figure S7. Proximity ligation assay for $TP53^{R273H}$ and BCAR1.

(A) Confocal microscopy images. H1299 cells were transfected with the indicated plasmids and subjected to proximity ligation assay (PLA) and immunofluorescence staining. Cells transfected with FLAG and/or MYC tag-containing plasmids serve as negative controls. The PLA signals (green), nuclei (blue), FLAG (red), and MYC (magenta) are shown. Scale bar represents 10 μm.

(B) Quantifications of the proximity ligated foci. The images obtained in (A) were used for calculation. Bars represent the mean fluorescence intensity of proximity ligated foci in each cell normalized by cell area ± SD. Eighteen or more cells were used for quantifications and indicated as dots. **P-value < 0.01. Statistical analysis of data was done by unpaired student's two-sided t-test.

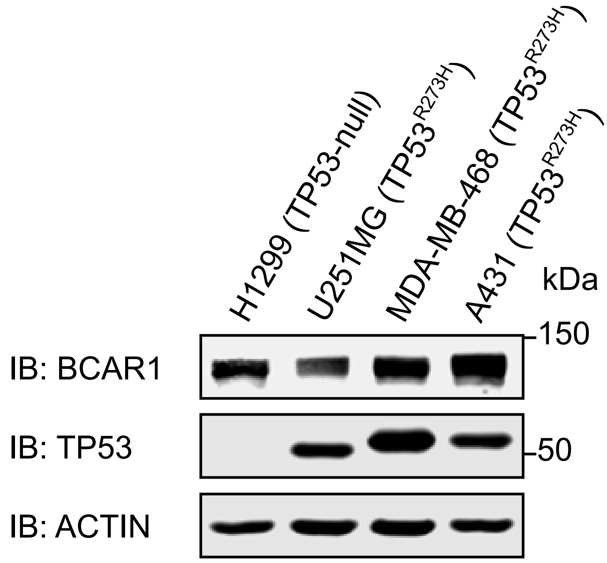

Supplementary Figure S8. Western blot analysis of $TP53^{R273H}$ and BCAR1.
Lysates of the indicated cell lines were analysed by Western blot.
