# Supplementary material for: Mutant TP53 interacts with BCAR1 to contribute to cancer cell invasion
Source: Br J Cancer. 2020 Nov 4;124(1):299–312. doi: 10.1038/s41416-020-01124-9 (PMC7782524; doi:10.1038/s41416-020-01124-9)
Supplement: Supplementary file 2 — Supplementary Tables [file 41416_2020_1124_MOESM2_ESM.docx]

**Supplementary Table 1. List of 151 proteins identified in our mass spectrometry screen.**

| **Gene symbols** |
| --- |
| \| ACAT2 \| CCT8 \| GNB2 \| LONP2 \| PPP6C \| SLC35F4 \| \| --- \| --- \| --- \| --- \| --- \| --- \| \| AIP \| CDRT15L2 \| GNL2 \| LRMP \| PRDX2 \| SMG5 \| \| AKAP10 \| CFL2 \| GPS1 \| MAPK1 \| PRDX3 \| SMG7 \| \| ALB \| CHD7 \| GRB2 \| MAST1 \| PSMC3 \| SPG20 \| \| ANKRD22 \| CLEC18A \| GRIA2 \| MC1R \| PSMC3IP \| TCP1 \| \| ANKRD54 \| CLTC \| GRN \| MCM5 \| PSMC4 \| THBS1 \| \| BAG2 \| CNP \| GSPT1 \| MDM2 \| PSMD2 \| TP53 \| \| BCAR1 \| COPS3 \| GTF3A \| NMT1 \| PSMD9 \| TPM4 \| \| BRCC3 \| CUL7 \| HSP90AA1 \| NRD1 \| PTGES3 \| TRAF4 \| \| C10ORF2 \| CUL9 \| HSP90AB1 \| NT5C2 \| RAB3C \| TRAP1 \| \| C19ORF62 \| CXORF38 \| HSPA1A \| NTHL1 \| RABEP1 \| TRIP12 \| \| C20ORF11 \| CYTH3 \| HSPA4L \| OAZ1 \| RABGAP1 \| TUFM \| \| C2CD2L \| DDX6 \| HSPA9 \| OLA1 \| RIC8B \| UBL4B \| \| C2ORF56 \| DECR1 \| HSPD1 \| OSBPL2 \| RNH1 \| UROD \| \| CAPN2 \| DNAJA1 \| HSPH1 \| OTOGL \| RPL14 \| USP11 \| \| CAPN3 \| DNAJA2 \| IGKV2-28 \| OTOP1 \| RPL23 \| USP7 \| \| CAPNS1 \| DPYSL3 \| IGKV2D-26 \| PCBP1 \| RPL5 \| YWHAQ \| \| CBX3 \| EEF2 \| IKBIP \| PCBP2 \| RPL6 \| YWHAZ \| \| CCDC42B \| EIF5A \| KANK1 \| PCCA \| RPS14 \| ZNF165 \| \| CCDC63 \| EPHA6 \| KAT5 \| PCNA \| RPS3 \| ZNF292 \| \| CCT2 \| ERBB2IP \| KATNB1 \| PCSK1 \| S100A10 \| ZNF721 \| \| CCT3 \| FAM135B \| KHDRBS1 \| PDPK1 \| S100A2 \|  \| \| CCT4 \| FASN \| KLB \| PGM1 \| SFTPD \|  \| \| CCT5 \| FBXW8 \| KPNA1 \| PLOD1 \| SKOR2 \|  \| \| CCT6A \| FHL2 \| KPNA3 \| PPP1CB \| SKP1 \|  \| \| CCT7 \| FIGNL1 \| LGALS1 \| PPP2CA \| SLC35A1 \|  \| |

**Supplementary Table 2. List of proteins identified under the cellular movement category.**

Among the proteins identified by mass spectrometry analysis, the proteins listed under the cellular movement category of the molecular and cellular functions in the Ingenuity Pathways Analysis are shown with the protein symbol. The expectation value obtained by mass spectrometry analysis using GPM software (http://gpmdb.thegpm.org) and the CRAPome value obtained from CRAPome database (http://www.crapome.org) are also shown.

| **Cellular movement**  **(17 proteins)** | | |
| --- | --- | --- |
| **Gene**  **symbol** | **Expectation**  **value log(e)** | **CRAPome**  **value** |
| CUL7 | -323.9 | 3/411 |
| MC1R | -237.9 | 0/411 |
| NRD1 | -84.6 | 8/411 |
| GRB2 | -42 | 7/411 |
| PDPK1 | -33.7 | 0/411 |
| S100A10 | -32.3 | 6/411 |
| MDM2 | -31.1 | 0/411 |
| THBS1 | -27.3 | 8/411 |
| FHL2 | -9 | 4/411 |
| S100A2 | -7 | 2/411 |
| GRN | -4 | 9/411 |
| TRAF4 | -4 | 8/411 |
| KANK1 | -2.1 | 3/411 |
| GRIA2 | -1.3 | 0/411 |
| BCAR1 | -1.2 | 2/411 |
| RABEP1 | -1.2 | 13/411 |
| SFTPD | -1 | 0/411 |
